# Diagnostic Accuracy Study of Intraoperative and Perioperative Serum Intact PTH Level for Successful Parathyroidectomy in 501 Secondary Hyperparathyroidism Patients

**DOI:** 10.1038/srep26841

**Published:** 2016-05-27

**Authors:** Lina Zhang, Changying Xing, Chong Shen, Ming Zeng, Guang Yang, Huijuan Mao, Bo Zhang, Xiangbao Yu, Yiyao Cui, Bin Sun, Chun Ouyang, Yifei Ge, Yao Jiang, Caixia Yin, Xiaoming Zha, Ningning Wang

**Affiliations:** 1 Department of Nephrology, First Affiliated Hospital of Nanjing Medical University, Nanjing, Jiangsu Province, 210029, People’s Republic of China; 2 Department of Epidemiology and Biostatistics, School of Public Health, Nanjing Medical University, Nanjing, Jiangsu Province, 211166, People’s Republic of China; 3 Department of General Surgery, First Affiliated Hospital of Nanjing Medical University, Nanjing, Jiangsu Province, 210029, People’s Republic of China

## Abstract

Parathyroidectomy (PTX) is an effective treatment for severe secondary hyperparathyroidism (SHPT); however, persistent SHPT may occur because of supernumerary and ectopic parathyroids. Here a diagnostic accuracy study of intraoperative and perioperative serum intact parathyroid hormone (iPTH) was performed to predict successful surgery in 501 patients, who received total PTX + autotransplantation without thymectomy. Serum iPTH values before incision (io-iPTH0), 10 and 20 min after removing the last parathyroid (io-iPTH10, io-iPTH20), and the first and fourth day after PTX (D1-iPTH, D4-iPTH) were recoded. Patients whose serum iPTH was >50 pg/mL at the first postoperative week were followed up within six months. Successful PTX was defined if iPTH was <300 pg/mL, on the contrary, persistent SHPT was regarded. There were 86.4% patients underwent successful PTX, 9.8% remained as persistent SHPT and 3.8% were undetermined. Intraoperative serum iPTH demonstrated no significant differences in two subgroups with or without chronic hepatitis. Receiver operating characteristic (ROC) curves showed that >88.9% of io-iPTH20% could predict successful PTX (area under the curve [AUC] 0.909, sensitivity 78.6%, specificity 88.5%), thereby avoiding unnecessary exploration to reduce operative complications. D4-iPTH >147.4 pg/mL could predict persistent SHPT (AUC 0.998, sensitivity 100%, specificity 99.5%), so that medical intervention or reoperation start timely.

Secondary hyperparathyroidism (SHPT) is a complex disorder associated with end-stage renal disease (ESRD), and is characterized by persistently elevated levels of serum intact parathyroid hormone (iPTH) and parathyroid hyperplasia^1^. SHPT patients present with various bone disorders, cardiovascular disease, and typical laboratory abnormalities. Parathyroidectomy (PTX) is necessary for severe SHPT patients who are resistant to pharmacological therapies^2^.

Human beings usually have four parathyroid glands. Successful PTX with all parathyroid glands removed should be performed in SHPT patients to avoid continuous residual gland stimulation in chronic kidney disease (CKD) environment. However, it is difficult to resect all parathyroid glands because of the existence of supernumerary and ectopic parathyroids. Persistent SHPT is reported to be 0.4% to 25% after PTX^3^,^4^,^5^.

Since iPTH is mainly metabolized in the liver and kidney^6^, the impairment of hepatic or renal function may prolong the breakdown of iPTH^7^,^8^,^9^. The half-life of iPTH was suggested to be about 2 minutes under normal kidney function^10^ and about 5 minutes under renal failure^8^. Although several studies have investigated the predictive value of intraoperative iPTH (io-iPTH) in SHPT patients^11^,^12^,^13^, the results are still controversial. Whether chronic hepatitis affects io-iPTH diagnostic accuracy in PTX patients remains unknown.

Here we assessed the predictive value of intraoperative and perioperative iPTH monitoring for successful PTX with autotransplantation in SHPT patients, to improve the success rate of PTX and reduce the complications caused by unnecessary exploration. Furthermore, perioperative iPTH monitoring could help physicians find persistent SHPT in an early stage after surgery and medical treatments or reoperation could be initiated in a timely manner. Also, the differences in io-iPTH declines among patients with or without chronic hepatic disease were also discussed.

## Results

### Participant characteristics

We enrolled 120 healthy controls (55 males and 65 females), 121 stage 5 CKD patients without severe SHPT (64 males and 57 females), and 501 severe SHPT patients (277 males and 224 females) from April 2011 to August 2015. The median baseline iPTH level of SHPT patients was 2132.1 pg/mL. Clinical characteristics and laboratory values in healthy controls and patients were shown in Table 1. Compared with healthy controls (n = 120), stage 5 CKD patients (n = 121) and PTX patients (n = 501) had lower hemoglobin (Hb), hematocrit (Hct), serum albumin (Alb), alanine amino transferase (ALT), aspartate amino transferase (AST), direct bilirubin (DBIL), and indirect bilirubin (IBIL) levels. Also these patients had higher blood pressure, serum alkaline phosphatase (ALP), iPTH, calcium, and phosphate levels. PTX patients were younger than stage 5 CKD patients without severe SHPT. Compared with the stage 5 CKD group, PTX patients had much more serious disorders in mineral metabolism, especially serum calcium, ALP, and iPTH levels.

**Figure 1:**
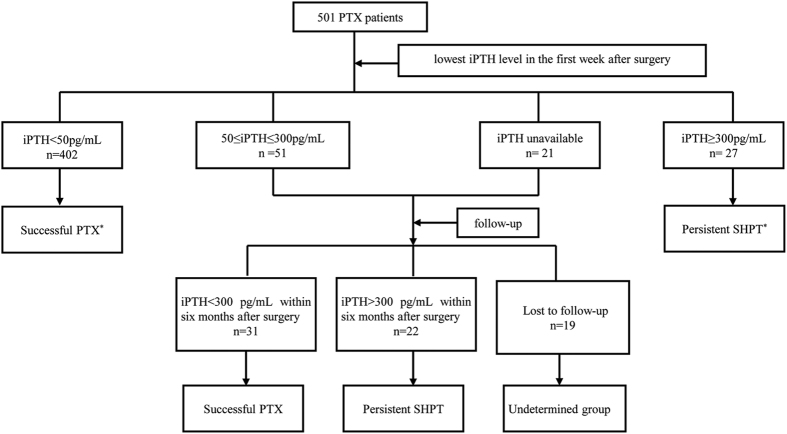
Flow chart of our study. *Patients not necessarily followed-up.

There were 433 (86.4%) patients who underwent successful PTX, 49 (9.8%) patients remained with persistent SHPT after surgery, while the other 19 (3.8%) patients lacking valid iPTH values were classified as the undetermined group (Fig. 1). Here we divided the PTX patients into four groups according to their baseline iPTH levels. The highest serum iPTH group (iPTH >2,500 pg/mL) was shown to be the youngest ones, and had lower serum glucose (Glu), higher Alb and ALP levels and parathyroid gland weights (Table 1).

### Number of resected parathyroid glands in PTX patients

**Figure 2:**
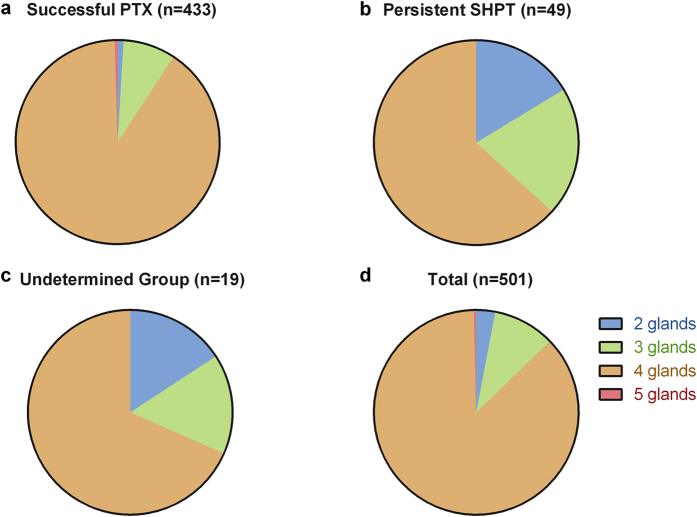
Number of parathyroid glands removed in PTX patients.

Operative exploration revealed that most patients had four parathyroid glands (435, 86.8%). Five glands were resected in 2 patients (0.4%) who turned out to have successful PTX. Three glands were identified in 49 patients (9.8%) and two glands were removed in 15 patients (3.0%) after careful exploration. In fact, the results in persistent SHPT group did not actually reflect their parathyroid gland numbers. Persistent SHPT patients tended to have more parathyroid glands than the recorded numbers (Fig. 2).

### Intraoperative and perioperative iPTH levels in PTX patients

Overall, the mean percentage reduction of serum iPTH at 10 and 20 minutes after PTX (io-iPTH10%, io-iPTH20%) in the successful PTX group were 86.8% and 90.6%, respectively, while the persistent SHPT group were 69.8% and 76.5%. We compared io-iPTH10% and io-iPTH20% between the successful PTX group and persistent SHPT group and found significant differences (both P<0.001).

**Figure 3:**
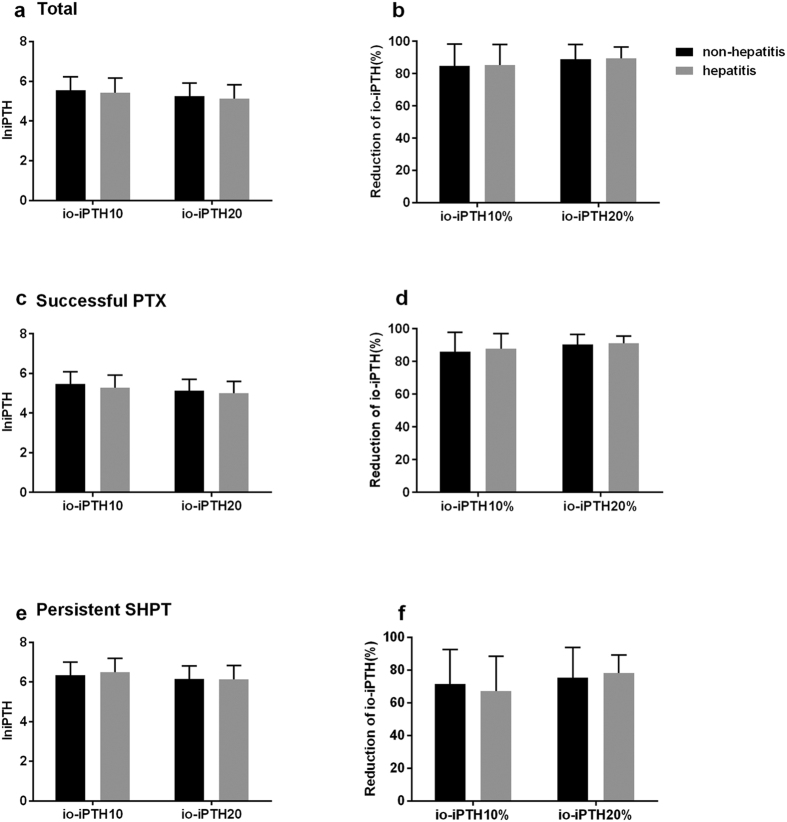
Levels of serum iPTH at 10 or 20 minutes after PTX (io-iPTH10, io-iPTH20) and their percentage reduction (io-iPTH10%, io-iPTH20%) in patients with or without hepatitis.

There were 204 hepatitis patients and 297 non-hepatitis patients in the PTX group. The baseline iPTH levels of patients with or without chronic hepatitis were 2,009.9 (1,393.5–2,801.4) pg/mL and 2,163.9 (1,577.9–3,009.9) pg/mL, respectively (*P* = 0.116). The levels of serum iPTH at 10 or 20 minutes after PTX (io-iPTH10, io-iPTH20) and their percentage reductions in these two subgroups also showed no significant differences (Fig. 3).

**Figure 4:**
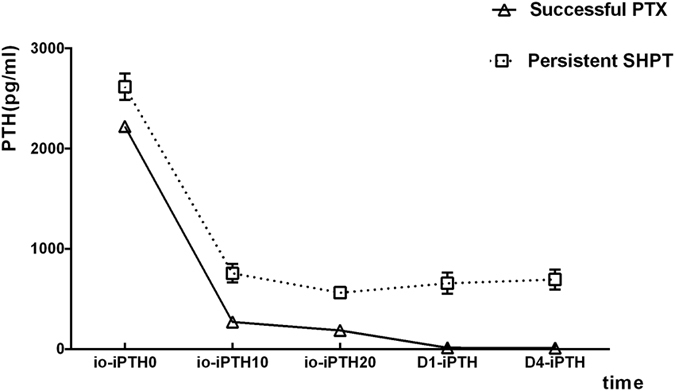
The change of serum iPTH level among different groups after PTX.

After surgery, serum iPTH in patients with successful PTX decreased from 2,081.5 (1,488.6–2,805.5) pg/mL to 9.3 (5.3–17.2) pg/mL on the first day (D1-iPTH) and further to 5.1 (2.5–13.2) pg/mL on the fourth day (D4-iPTH). Meanwhile, the serum iPTH levels in the persistent SHPT patients were 2,787.0 (1,730.4–3,381.5) pg/mL (baseline), 489.9 (247.0–897.7) pg/mL (D1-iPTH), and 546.1 (271.2–900.6) pg/mL (D4-iPTH), respectively (Fig. 4).

### Intraoperative and perioperative iPTH cutoff values for the prediction of successful PTX or persistent SHPT

**Figure 5:**
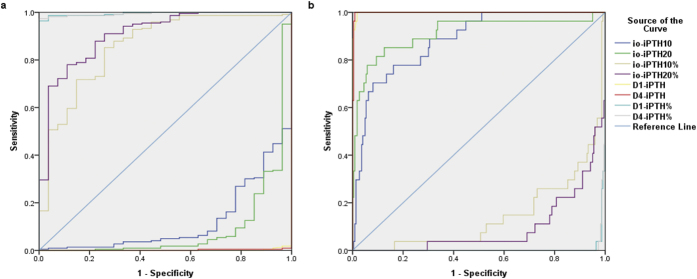
The ROC curves for predicting of successful PTX or persistent SHPT.

The cutoff values of intraoperative and perioperative iPTH were listed in Table 2. Sensitivity and specificity were obtained from the ROC analyses (Fig. 5). It showed that the criterion of an 82.9% decline in io-iPTH10% could predict the success of surgery (area under the curve [AUC] 0.857, sensitivity 85.5%, and specificity 73.1%). In addition, 88.9% decline of io-iPTH20% was effective to predict successful PTX (AUC 0.909, sensitivity 78.6%, and specitivity 88.5%). D1-iPTH could predict persistent SHPT with a cutoff value of 100.5 pg/mL. AUC, sensitivity, and specitivity of this criterion were 0.999, 100% and 98.6% respectively. When D4-iPTH was used to predict persistent SHPT, the cut-off value was 147.4 pg/mL, the AUC was 0.998, sensitivity was 100%, and specificity was 99.5%.

### Diagnostic accuracy of intraoperative and perioperative iPTH monitoring

Io-iPTH20 decreased >88.9% from baseline in 317 PTX patients and, among them 297 patients underwent successful PTX. On the contrary, io-iPTH20 dropped <88.9% from baseline in 158 patients, whereas 40 patients had persistent SHPT (Table 3). The positive predictive value (PPV) and negative predictive value (NPV) of this criterion were 97.1% (95% CI 0.947 to 0.986) and 26.5% (95% CI 0.204 to 0.323) respectively.

D4-iPTH levels were >147.4pg/mL in 40 patients and, among them, 36 patients had persistent SHPT after surgery. On the contrary, D4-iPTH dropped to <147.4pg/mL in 362 patients, whereas 359 patients had successful PTX (Table 3). The PPV and NPV of this criterion were 90.0% (95% CI 0.763 to 0.972) and 99.2% (95% CI 0.976 to 0.998) respectively.

## Discussion

Successful PTX could improve symptoms effectively and reduce the risk of all-cause and cardiovascular mortality in severe SHPT patients^14^. All parathyroid glands must be removed completely during the surgery for successful PTX. SHPT patients had a higher risk of supernumerary parathyroid glands^5^,^15^. Our results demonstrated that the rate of resected 2, 3, 4, or 5 parathyroids were 3.0%, 9.8%, 86.8% and 0.4%, respectively. Actually, the exact number of parathyroids in persistent SHPT patients was more than we recorded.

Parathyroid glands originate from the endoderm of the third and fourth pharyngeal pouches. Based on the anatomic and developmental characteristics of parathyroid glands, surgical procedures for SHPT patients include subtotal parathyroidectomy (sPTX) and total parathyroidectomy (tPTX) with or without autotransplantation (AT). Ectopic parathyroid could exist in any location of the migration path including the intrathyroid, carotid sheath, thymus, and upper mediastinum^13^. According to previous data, the frequency of ectopic parathyroid glands was about 15% in SHPT patients^16^,^17^,^18^. Supernumerary and ectopic parathyroid glands make it difficult to perform successful PTX. Furthermore, excess exploration carries a higher risk of surgical complications including nerve injuries and bleeding, also increase perioperative period mortality. Thus, the confirmation of the complete removal of parathyroid glands during the surgery is required. Our results demonstrated that io-iPTH monitoring was a useful tool for predicting successful PTX in SHPT patients (Fig. 5). We showed more than an 82.9% decrease of io-iPTH10 could predict a complete parathyroid gland excision. As Fig. 4 shows, iPTH levels decreased gradually during the surgery. If io-iPTH10 failed to reach the above criterion, we recommend a criterion of >88.9% decrease in io-iPTH20. Also, we studied the perioperative iPTH values and found that D4-iPTH exceeding 147.4 pg/mL could effectively predict the persistence of SHPT after surgery. Patients not achieving this criterion are suggested to be followed-up closely, and medication intervention should be started. Another operation may even be necessary.

Studies identified the liver as a major extra renal site of iPTH metabolism^6^,^19^. Chronic hepatitis, which is mainly caused by hepatitis B virus (HBV) and hepatitis C virus (HCV), could be transmitted via infected blood products. Among patients receiving maintenance dialysis, the prevalence of HBV ranges from 1.3–14.6% and the prevalence of HCV is 0.7–18.1%^20^,^21^. To our knowledge, the influence of chronic hepatitis on intraoperative serum iPTH values in PTX patients has not been studied previously. We showed that hepatitis had no effect on intraoperative iPTH values. Perhaps this may be explained by extremely abnormal hepatic functions being a contraindication of surgery. Our patients all had normal serum ALT, AST, DBIL, IBIL levels and prothrombin times even if they had a chronic hepatitis history.

Hiramitsu *et al.*^22^ studied the predictive value of io-iPTH in 226 PTX patients and found that an iPTH value of <60 pg/mL on postoperative day 1 (sensitivity and specificity not mentioned) could predict successful parathyroidectomy and a 70% io-iPTH drop from the baseline at 10 minutes after surgery (sensitivity 97.5%, specificity 52.2%) could determine sufficient parathyroid gland removal. Compared with Hiramitsu’s research, the sensitivity and specificity of our criterion were higher, although there were some similarities. Our sample size was larger than theirs (501 vs 226). Further, we reported novel findings that there was no influence of chronic hepatitis on intraoperative iPTH values. In addition, we suggested that D4-iPTH >147.4 pg/mL (sensitivity 100%, specificity 99.5%) as a criterion of persistent SHPT could help physicians made an accurate decision to start medical treatments in a timely manner.

In conclusion, our results showed that chronic hepatitis had no effect on intraoperative iPTH monitoring. More than 88.9% of io-iPTH20% was effective to predict the success of surgery (sensitivity 78.6%, specificity 88.5%) and D4-iPTH >147.4 pg/mL was effective to predict persistent SHPT (sensitivity 100%, specificity 99.5%). Intraoperative iPTH monitoring could help surgeons make decisions to stop exploration in a timely manner, which will obviously reduce the risk of bleeding and nerve injuries obviously. Perioperative iPTH monitoring could effectively predict the persistence of SHPT after surgery and the patients can start medical intervention or reoperation as soon as possible.

## Concise Methods

### PTX patients

We enrolled 501 PTX patients including 277 men and 224 women who received total parathyroidectomy with forearm autotransplantation (tPTX + AT) without thymectomy in our hospital from April 2011 to August 2015. All data were collected retrospectively. PTX was performed in severe SHPT patients who failed to respond to medical therapy^2^. Our surgical indications included: persistent serum iPTH >800 pg/mL; hypercalcemia and/or hyperphosphatemia that could not be controlled by medical therapy; obvious clinical manifestations such as bone pain, pruritus, ectopic calcification or fracture; and at least one enlarged parathyroid gland discovered by ultrasound or a radiopharmaceutical technetium-99m-methoxyisobutylisonitrile (99mTc-MIBI) scan. None of the patients took vitamin D analogs and calcimimetics.

### Healthy and stage 5 CKD group

We also included 120 healthy volunteers and 121 stage 5 CKD patients without severe SHPT.

All clinical investigations were conducted according to the 2008 Declaration of Helsinki and good clinical practice guidelines. Written informed consent was obtained from all the subjects. The study protocols were approved by the Research Ethics Committee of the First Affiliated Hospital of Nanjing Medical University, People’s Republic of China.

### Surgical procedure

Preoperative evaluations included neck ultrasonography and parathyroid scintigraphy (99mTc-MIBI) for demonstrating the number, size, and location of parathyroid glands. Pulmonary functions, cardiac function, routine blood tests, and coagulation tests (including prothrombin time) were conventionally conducted before surgery so that contraindications would be discovered. TPTX + AT without thymetomy was performed routinely under general anesthesia in all SHPT patients. All operations were performed by the same surgeon. Bilateral neck examinations were carefully performed to make sure all hyperplastic parathyroid glands were resected.

Intraoperative frozen section analysis was routinely adopted to verify that the resected specimen was parathyroid tissue. The selected diffuse hyperplasia parathyroid fragment was cut into slices about 1 mm^3^ and 8 slices were transplanted into forearm muscles without an arteriovenous fistula for hemodialysis. After surgery, pathological sections were examined carefully.

### Intraoperative and perioperative iPTH monitor

Venous blood was drawn before the incision to determine the baseline io-iPTH (io-iPTH0). After resection of all explored parathyroid glands, io-iPTH levels were assayed at 10 min (io-iPTH10) and 20 min (io-iPTH20) after excision. Perioperative iPTH levels were determined on the first and fourth days after surgery (D1-iPTH, D4-iPTH). Serum iPTH levels were measured using a UniCel DxI800 Access Immunoassay System (Beckman Coulter, Inc., Fullerton, CA, U.S.). The recommended range of serum iPTH is 12~88 pg/mL among healthy controls. The rate of decrease in io-iPTH measured after resection compared to baseline levels were calculated and recorded (io-iPTH10%, io-iPTH20%, D1-iPTH%, and D4-iPTH%).

### Definition of successful PTX and persistent SHPT

According to previous studies, serum iPTH levels detected at the first postoperative week <300 pg/mL was the criterion of successful PTX^13^. Here we adopted a stricter criterion. Patients with serum iPTH<50 pg/mL at the first postoperative week were classified as successful PTX group. Patients with serum iPTH >50 pg/mL at the first postoperative week examination were followed up to verify the effect of surgery. Depending on serum iPTH values within six months, patients with iPTH <300 pg/mL were regarded as the successful PTX group, and those whose iPTH were >300 pg/mL were classified as persistent SHPT. Patients without valid iPTH values after surgery were classified as the undetermined group.

### Statistical analysis

All statistical analyses were performed using the Statistical Package for the Social Sciences (SPSS) version 20.0 (SPSS Inc., Chicago, IL, U.S.). Continuous variables were presented as mean ± SD or median (interquartile range), and categorical variables were presented as number and proportion. Differences between groups were compared using an independent samples t or Wilcoxon rank sum test for continuous variables and a chi-squared or Fisher’s exact test for categorical variables. *P* < 0.05 was considered statistically significant. Receiver operating characteristic (ROC) curves were used to identify the cutoff value for prediction of surgical success and persistent SHPT. Diagnostic accuracy was expressed through sensitivity, specificity, and the area under the ROC curve (AUC). The Standard Reporting for Diagnostic (STARD) studies were used here.

Patients were designated as true positive (TP), true negative (TN), false positive (FP), and false negative (FN) (Table 3). The PPV and NPV were calculated.

## Additional Information

**How to cite this article**: Zhang, L. *et al.* Diagnostic Accuracy Study of Intraoperative and Perioperative Serum Intact PTH Level for Successful Parathyroidectomy in 501 Secondary Hyperparathyroidism Patients. *Sci. Rep.* **6**, 26841; doi: 10.1038/srep26841 (2016).

## Figures and Tables

**Table 1:** Clinical characteristics and laboratory results.

	Control (n = 120)	Stage 5 CKD patients without PTX (n = 121)	PTX						P*
			PTH < 800 (n = 25)	800 ≤ PTH < 1500 (n = 97)	1500 ≤ PTH < 2500 (n = 194)	PTH ≥ 2500 (n = 185)	P#	Total (n = 501)	
Demographics									
Age	48.1 ± 13.7	49.9 ± 13.5	49.0 ± 9.0	49.0 ± 11.6	46.7 ± 11.3	43.0 ± 11.2	<0.001	45.9 ± 11.4	0.004
Male/Female	55/65	64/57	17/8	58/39	92/102	110/75	0.035	277/224	0.174
Systolic pressure (mmHg)	124.3 ± 18.2	149.6 ± 25.5	142.7 ± 21.0	142.1 ± 26.8	139.2 ± 19.2	139.4 ± 20.5	0.635	140.0 ± 21.4	<0.001
Diastolic pressure (mmHg)	78.3 ± 10.7	88.9 ± 12.5	86.4 ± 11.3	86.6 ± 12.5	85.6 ± 12.5	85.8 ± 11.7	0.921	85.9 ± 12.1	<0.001
Dialysis mode, n (%)									
Predialysis	120 (100%)	39 (32.2%)	0 (0.0)	0 (0.0)	0 (0.0)	0 (0.0)	NA	0 (0.0)	<0.001
Haemodialysis	0 (0.0)	48 (39.7%)	25 (100%)	87 (89.7%)	181 (93.3%)	173 (93.5%)	0.305	466 (93.0%)	<0.001
Peritoneal dialysis	0 (0.0)	34 (28.1%)	0 (0.0)	10 (10.3%)	13 (6.7%)	12 (6.5%)	0.305	35 (7.0%)	<0.001
Dialysis vintage (m)	0 (0.0–0.0)	7.0 (0.0–36.0)	96.0 (78.0–144.0)	84.0 (48.0–108.0)	84.0 (60.0–120.0)	84.0 (66.0–120.0)	0.021	84.0 (60.0–120.0)	<0.001
Cause of ESRD, n (%)									
Glomerulonephritis	0 (0.0)	73 (60.3%)	22 (88.0%)	86 (88.7%)	180 (92.8%)	173 (93.5%)	0.31	461 (92.0%)	<0.001
Diabetic nephropathy	0 (0.0)	19 (15.7%)	2 (8.0%)	2 (2.1%)	1 (0.5%)	1 (0.5%)	0.008	6 (1.2%)	<0.001
Hypertensive nephropathy	0 (0.0)	6 (5.0%)	1 (4.0%)	0 (0.0)	1 (0.5%)	2 (1.1%)	0.221	4 (0.8%)	0.001
Polycystic kidney disease	0 (0.0)	9 (7.4%)	1 (4.0%)	6 (6.2%)	7 (3.6%)	5 (2.7%)	0.543	19 (3.8%)	0.010
Other	0 (0.0)	15 (12.4%)	2 (8.0%)	3 (3.1%)	5 (2.6%)	1 (0.5%)	0.079	11 (2.2%)	<0.001
Laboratory values									
Hemoglobin (g/l)	143.5 ± 15.6	91.5 ± 20.5	108.6 ± 23.0	104.3 ± 19.7	102.2 ± 18.0	100.7 ± 20.1	0.186	102.4 ± 19.4	<0.001
Hematocrit (%)	43.1 ± 4.3	27.9 ± 6.2	33.6 ± 7.3	32.3 ± 7.1	31.8 ± 5.4	31.5 ± 6.3	0.405	31.9 ± 6.2	<0.001
Glucose (mmol/l)	5.4 ± 0.7	5.3 ± 2.7	5.1 ± 3.5	4.4 ± 0.9	4.3 ± 1.4	4.2 ± 0.7	0.010	4.3 ± 1.3	<0.001
Creatinine (μmol/l)	71.7 ± 16.1	861.7 ± 352.4	970.9 ± 307.7	934.0 ± 273.2	875.6 ± 281.2	833.1 ± 262.5	0.008	876.0 ± 276.7	<0.001
Urea (mmol/l)	5.4 ± 1.4	24.2 ± 9.8	23.5 ± 9.3	22.1 ± 7.9	22.3 ± 8.1	22.2 ± 8.7	0.893	22.3 ± 8.3	<0.001
Albumin (g/l)	47.3 ± 3.1	36.9 ± 5.1	39.8 ± 5.5	39.1 ± 4.9	38.6 ± 4.8	37.5 ± 4.0	0.010	38.3 ± 4.6	<0.001
ALT (U/L)	15.1 (11.0–23.8)	12.2 (9.2–18.2)	14.8 (11.2–27.7)	12.5 (8.5–18.3)	10.1 (7.7–18.6)	8.9 (6.4–15.5)	<0.001	10.3 (7.2–17.4)	<0.001
AST (U/L)	24.3 (19.3–28.5)	16.1 (13.5–20.1)	19.2 (12.2–23.6)	14.4 (11.2–19.9)	14.4 (11.4–19.2)	13.7 (10.2–18.9)	0.068	14.4 (10.9–19.4)	<0.001
DBIL (μmol/l)	3.3 (2.5–4.6)	1.7 (1.1–2.3)	1.7 (1.0–2.2)	1.7 (1.3–2.5)	1.6 (1.2–2.3)	1.6 (1.2–2.3)	0.870	1.6 (1.2–2.3)	<0.001
IBIL (mol/l)	6.9 (5.5–9.6)	3.3 (2.6–4.6)	3.5 (2.5–4.6)	3.5 (2.6–4.8)	3.7 (3.0–5.0)	3.7 (2.8–4.7)	0.396	3.6 (2.8–4.8)	<0.001
Calcium (mg/dl)	9.4 ± 0.4	9.0 ± 1.2	10.7 ± 1.0	10.2 ± 0.9	10.3 ± 0.9	10.1 ± 0.9	0.014	10.2 ± 0.9	<0.001
Phosphorus (mg/dl)	3.7 ± 0.5	6.3 ± 2.0	6.9 ± 1.7	6.8 ± 1.6	6.8 ± 1.6	6.6 ± 1.6	0.572	6.7 ± 1.6	<0.001
ALP (μ/l)	73.8 (62.7–86.8)	86.9 (70.6–106.2)	93.6 (79.4–135.2)	159.4 (117.3–251.3)	345.8 (218.3–666.4)	754.1 (417.2–1195.4)	<0.001	359.3 (177.4–806.2)	<0.001
lnALP	4.3 ± 0.3	4.5 ± 0.4	4.6 ± 0.4	5.1 ± 0.5	6.0 ± 0.8	6.5 ± 0.8	<0.001	5.9 ± 0.9	<0.001
iPTH (pg/ml)	34.5 (27.1–47.3)	229.3 (115.2–361.1)	708.8 (623.0–751.6)	1154.0 (992.9–1343.8)	1979.0 (1741.7–2219.2)	3215.7 (2793.2–3460.3)	<0.001	2132.1 (1510.8–2945.8)	<0.001
lniPTH	3.6 ± 0.4	5.3 ± 0.9	6.5 ± 0.2	7.0 ± 0.2	7.6 ± 0.1	8.1 ± 0.2	<0.001	7.6 ± 0.5	<0.001
Weight of parathyroid gland	NA	NA	2.6 (1.8–4.4)	3.3 (2.3–4.7)	3.8 (2.7–5.3)	4.3 (3.1–5.9)	<0.001	3.9 (2.6–5.3)	<0.001

Data are mean ± standard deviation (SD), or numbers and percentages, or median (25th–75th percentile), as appropriate. Significance between two groups were obtained from Independent-Samples t test or Wilcoxon’s rank sum test for continuous variables and Chi-square test or Fisher’s exact test for categorical variables. Significance between four groups were obtained from Kruskal-Wallis test, one-way ANOVA, or Chi-square test.

ESRD, end stage renal disease; ALT, alanine amino transferase; AST, aspartate amino transferase; DBIL, direct bilirubin; IBIL, indirect bilirubin; ALP, alkaline phosphatase; iPTH, intact parathyroid hormone; PTX, parathyroidectomy.

^#^Difference among PTX groups with different iPTH levels.

^*^Difference among controls, stage 5 CKD patients, and PTX patients.

**Table 2:** Results of ROC curve.

		AUC	Cutoff value	Sensitivity	Specificity
Successful PTX	io-iPTH10	0.119	45.1	100%	0
	io-iPTH10%	0.857	82.9%	85.5%	73.1%
	io-iPTH20	0.090	40.3	100%	0
	io-iPTH20%	0.909	88.9%	78.6%	88.5%
	D1-iPTH	0.001	−0.1	100%	0
	D1-iPTH%	0.997	96.5%	97.3%	100%
	D4-iPTH	0.002	−1.0	100%	0
	D4-iPTH%	0.996	95.5%	97.7%	100%
Persistent SHPT	io-iPTH10	0.881	485.7	69.2%	91.8%
	io-iPTH10%	0.143	3.2%	100%	0.9%
	io-iPTH20	0.910	291.2	84.6%	87.3%
	io-iPTH20%	0.091	35.6%	100%	0
	D1-iPTH	0.999	100.5	100%	98.6%
	D1-iPTH%	0.003	−17.4%	100%	0
	D4-iPTH	0.998	147.4	100%	99.5%
	D4-iPTH%	0.004	4.1%	100%	0

AUC: area under the curve; PTX: parathyroidectomy.

**Table 3:** Diagnostic accuracy of io-iPTH monitoring for successful PTX and perioperative iPTH monitoring for persistent SHPT.

	Successful PTX	Persistent SHPT	Total
Io-iPTH20% >88.9%	TP297	FP9	306
Io-iPTH20% <88.9%	FN111	TN40	151
Total	408^1^	49	457^2^
D4-iPTH >147.4pg/mL	TP36	FP4	40
D4-iPTH <147.4pg/mL	FN3	TN359	362
Total	39^4^	363^3^	402^5^

True positive (TP), true negative (TN), false positive (FP), false negative (FN). The percentage reduction of io-iPTH20 from basal iPTH levels (io-iPTH20%).

^1^25 successful PTX patients missing io-iPTH20% value.

^2^25 Persistent SHPT patients missing io-iPTH20% value and 19 patients in undetermined group.

^3^70 Successful PTX patients missing D4-iPTH value.

^4^10 Persistent SHPT patients missing D4-iPTH value.

^5^70 Successful PTX patients and 10 Persistent SHPT patients missing D4-iPTH value, and 19 patients in undetermined group.
